# Bedside microdialysis for detection of early brain injury after out-of-hospital cardiac arrest

**DOI:** 10.1038/s41598-021-95405-9

**Published:** 2021-08-05

**Authors:** Simon Mölström, Troels Halfeld Nielsen, Carl H. Nordström, Axel Forsse, Sören Möller, Sören Venö, Dmitry Mamaev, Tomas Tencer, Henrik Schmidt, Palle Toft

**Affiliations:** 1 Department of Anesthesiology and Intensive Care, Odense University Hospital, J. B. Winsløws Vej 4, 5000 Odense, Denmark; 2 Department of Neurosurgery, Odense University Hospital, Odense, Denmark; 3 OPEN, Open Patient Data Explorative Network, Odense University Hospital, Odense, Denmark; 4 Department of Clinical Research, University of Southern Denmark, Odense, Denmark

**Keywords:** Hypoxic-ischaemic encephalopathy, Cardiology, Biomarkers

## Abstract

Bedside detection and early treatment of lasting cerebral ischemia may improve outcome after out-of-hospital cardiac arrest (OHCA). This feasibility study explores the possibilities to use microdialysis (MD) for continuous monitoring of cerebral energy metabolism by analyzing the draining cerebral venous blood. Eighteen comatose patients were continuously monitored with jugular bulb and radial artery (reference) MD following resuscitation. Median time from cardiac arrest to MD was 300 min (IQR 230–390) with median monitoring time 60 h (IQR 40–81). The lactate/pyruvate ratio in cerebral venous blood was increased during the first 20 h after OHCA, and significant differences in time-averaged mean MD metabolites between jugular venous and artery measurements, were documented (p < 0.02). In patients with unfavorable outcome (72%), cerebral venous lactate and pyruvate levels remained elevated during the study period. In conclusion, the study indicates that jugular bulb microdialysis (JBM) is feasible and safe. Biochemical signs of lasting ischemia and mitochondrial dysfunction are frequent and associated with unfavorable outcome. The technique may be used in comatose OHCA patients to monitor biochemical variables reflecting ongoing brain damage and support individualized treatment early after resuscitation.

## Introduction

Survival rates around 50% are reported in comatose patients treated with hypothermia in an Intensive Care Unit (ICU) after out-of-hospital cardiac arrest (OHCA)^1–3^. The ICU mortality is essentially due to the primary hypoxic-ischemic cerebral insult followed by secondary brain injury, including delayed cerebral hypoperfusion and impaired microcirculation as well as ischemia–reperfusion injury^4,5^. Secondary injury is a significant determinant of neurologic outcome, and alleviating its deleterious effects is a mainstay of post-cardiac arrest management^6^. However, our knowledge regarding cerebral pathophysiological and biochemical events in the initial period after restored circulation are limited and there is a need of bedside monitoring methods to identify potentially injurious processes^7^.

Microdialysis is an established technique for monitoring regional cerebral energy metabolism during neurocritical care^7^. Under non-neurosurgical conditions, it is problematic to insert an intracerebral catheter and a limited number of patients have been studied following resuscitation after cardiac arrest^8–10^. Supported by previous experimental animal studies, our group has recently shown that jugular bulb microdialysis (JBM) is representative of overall cerebral energy metabolism and may be used during cardiac surgery and extracorporeal oxygenation^11–13^. This feasibility study was designed to investigate whether bedside JBM reflects secondary brain injury after OHCA, and may be implemented as a clinical tool with implications for early prognosis and individualized treatment improving patient outcome. Therefore, we tested the hypothesis of whether the lactate/pyruvate (LP) ratio monitored in the cerebral venous outflow changed over time, reflecting cerebral metabolism after cardiac arrest, and hence was different from corresponding LP ratio observed in arterial blood.

The primary objective was to compare time-averaged means of microdialysis parameters (intervals of 12 h) of the jugular venous and the arterial blood during post-resuscitation care. Secondary objectives of clinical interest were to compare (1) neuro-metabolic patterns between patients with unfavorable and favorable neurological outcome (2) total duration of cerebral desaturation and clinical outcome.

## Methods

### Trial design

This single-center prospective feasibility study enrolled patients at Odense University Hospital, Denmark, from May 2018 to October 2019. The Danish Regional Committee on Health and Research Ethics and Danish Data Protection Agency approved the study under Project Number: S-20130166. In addition, the trial was registered at ClinicalTrials.gov, identifier: NCT03095742 (17/03/2017).

### Patients

Patients with sustained return of spontaneous circulation (ROSC) after OHCA were eligible for inclusion if complying with the following criteria: age ≥ 18 years, OHCA of presumed cardiac cause, score ≤ 8 on the Glasgow Coma Scale (GCS). Exclusion criteria were: unwitnessed asystole, cardiogenic shock with use of cardiac assist devices, suspected or confirmed ischemic stroke, intracranial bleeding, and known limitations in therapy. The prehospital study dataset complied with the Utstein definitions^14^. In accordance with national requirements and the principles of the Declaration of Helsinki, informed consent was obtained from next of kin.

### Post-resuscitation procedure

Eighteen unconscious patients with sustained ROSC were admitted to the cardiac intensive care unit following OHCA. JBM and near-infrared spectroscopy monitoring were the sole modifications from international clinical treatment guidelines for comatose OHCA patients^15^. Immediate angiography and percutaneous coronary intervention (PCI), when indicated, was performed in all resuscitated patients. Targeted temperature management (TTM) was commenced at the time of ICU admission targeting 36.0 °C for 24 h followed by controlled rewarming at a rate of 0.5 °C/h. A mean arterial pressure (MAP) > 65 mmHg was targeted. Mechanical ventilation was adjusted to achieve normocapnia (PaCO_2_ of 4.5–6.0 kPa) and oxygenation was maintained in the range of 13–14 kPa. Blood-glucose level was strictly maintained between 6 and 10 mmol/l.

### Neuromonitoring

JBM was initiated after ICU admission and continued for 96 h or until arousal utilizing identical techniques as in previous publications^13,16^. Intravenous MD catheters (CMA 67 IV, MDialysis AB, Stockholm, Sweden) were inserted in one jugular vein and one peripheral artery. The dominant jugular vein was accessed by retrograde insertion of a MD catheter (130 mm), through a 16 G intravenous catheter, with the tip placed in the jugular bulb under ultrasound guidance. The optimal positioning of the MD catheter tip corresponds to the anatomical landmark at level of mastoid. In accordance with previous studies, the correct positioning of the jugular bulb catheter tip was confirmed on cranial computed tomography (CT) scan (Supplementary information Figs. 1 and 2).

The catheters were perfused from a MD pump (CMA 106, MDialysis AB, Stockholm, Sweden) MD at a flow-rate of 0.3 μL/min with Ringers Acetate and dalteparin sodium (25 IU/mL). Samples of energy-related metabolites (lactate, pyruvate, glucose, glutamate, glycerol), were collected by the hour in microvials and analyzed utilizing enzymatic photometric techniques (Iscus, MDialysis AB, Stockholm, Sweden). With this perfusion rate the relative recovery has in cerebral tissue, been shown to be about 70% for the variables studied. When placed in a fluid (arterial or venous blood), the relative recovery is expected to be considerably higher. In the present study, the accuracy of the MD catheter was evaluated by studying the correlation and agreement between systemic blood lactate (Lac_sys_) and MD arterial lactate (Lac_MD-Art_).

Bilateral regional cerebral oxygen saturation (rSO_2_) was monitored continuously every hour for 96 h or until arousal (Somanetics INVOS Cerebral Oximeter system) with a pre-defined desaturation threshold of 50%^17^. The clinicians did not change routine clinical practice based on either bedside rSO_2_ or JBM monitoring. Routine intermittent electroencephalography (EEG) was performed after rewarming in patients who were still comatose.

### JBM reference values and classification of ischemia

**Figure 1:**
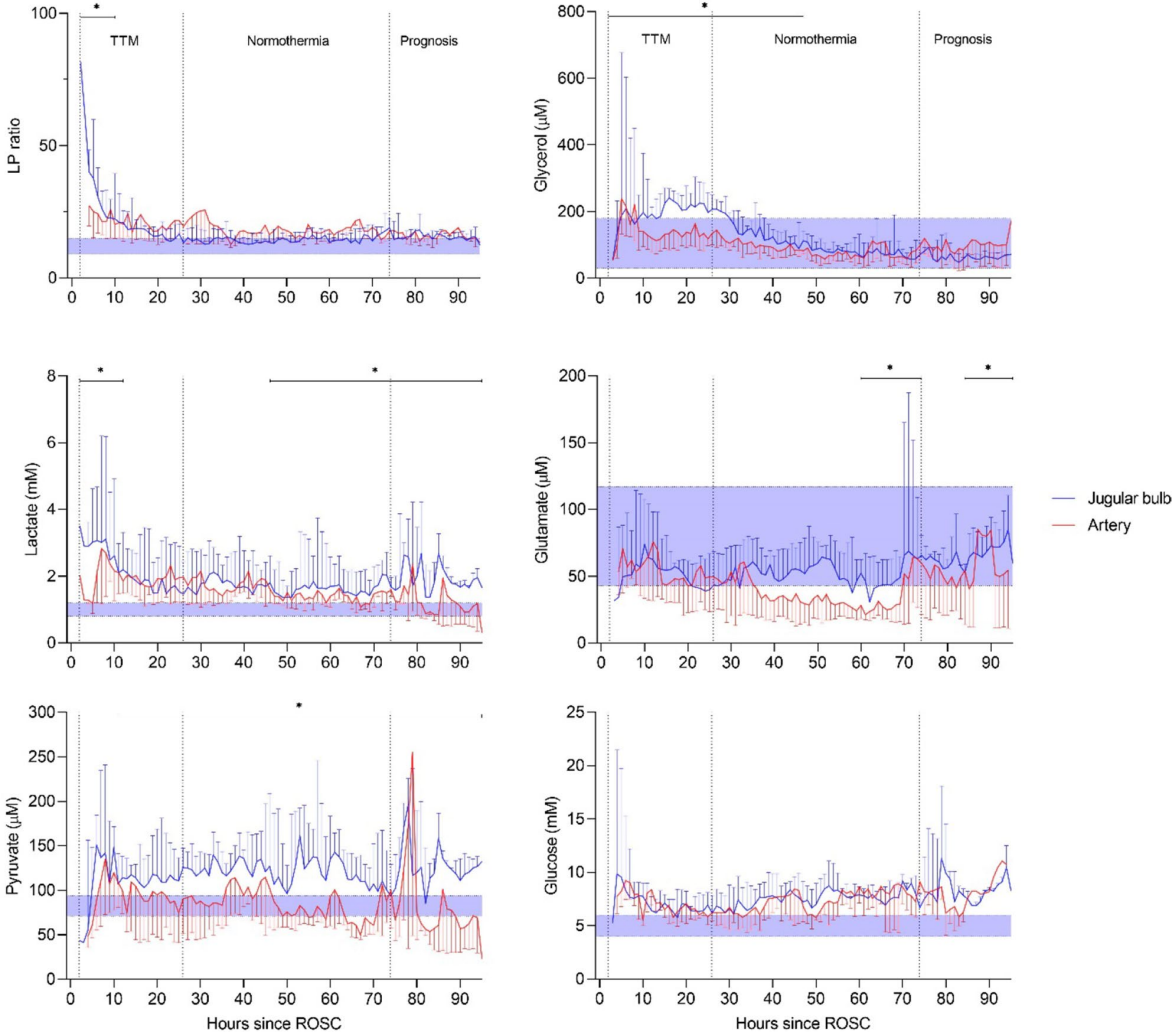
Median (IQR). Microdialysis parameters of the jugular venous and arterial blood during post-resuscitation care in patients with unfavorable outcome (n = 13). The difference between time-averaged means (in intervals of 12 h) of LP ratio, lactate, pyruvate, glycerol and glutamate of the jugular venous and the arterial blood was significant during post-resuscitation care (*p < 0.02) when using mixed effects models. Normal reference values are displayed (shaded area) in the graphs for each JBM variable.

**Figure 2:**
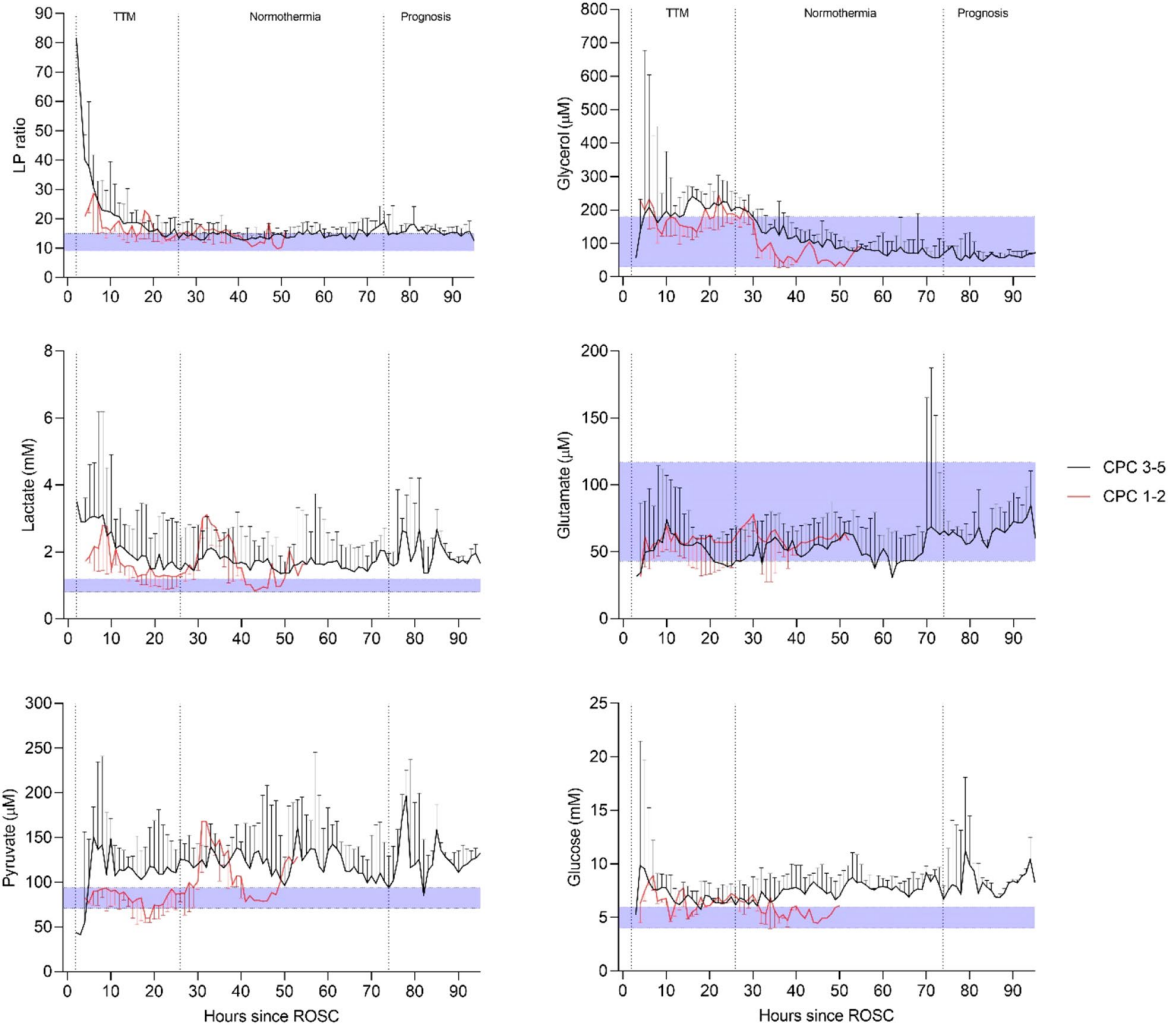
Median (IQR). Jugular bulb microdialysis parameters during post-resuscitation care in patients with unfavorable outcome (CPC 3–5, n = 13) compared to patients with favorable outcome (CPC 1–2, n = 5). The difference between time-averaged means (intervals of 12 h) of LP ratio, lactate, pyruvate, glycerol, glutamate and glucose between outcome groups, was not significant during post-resuscitation care when using mixed effects models. Normal reference values are displayed (shaded area) in the graphs for each JBM variable.

The definition of normal levels of the studied variables (lactate, pyruvate, glucose, glycerol, glutamate) in human jugular vein blood was based on JBM reference values obtained in anesthetized patients undergoing elective cardiac bypass surgery, indicated in Figs. 1 and 2^13^. Preoperative metabolite concentrations may approximate normal values relevant for an OHCA population. We proposed the following pathological thresholds for JBM at 0.3 µL/min with cutoff values for JBM (mean ± 2SD). Biochemical definitions of ischemia and mitochondrial dysfunction were based on principles obtained from intracerebral microdialysis^18,19^. In the present study LP ratio > 16 at pyruvate < 70 µM was classified as ischemia, and a pattern with LP ratio > 16 at pyruvate > 70 µM was classified as mitochondrial dysfunction.

### Neurologic prognostication

Active treatment continued until 72 h after TTM, with the exception of patients with brain death, status myoclonus or refractory shock with multiple organ dysfunctions. Patients who remained unconscious despite cessation of sedation were assessed with a combination of neurologic examination, EEG, somatosensory-evoked potential (SSEP) and CT of the brain. EEGs were classified into highly malignant if following patterns were present: suppression, suppression with periodic discharges, burst-suppression^20^. The clinicians performing the neurological prognostication were blinded to microdialysis variables. Decisions to withdraw life-supporting therapy were made by a multidisciplinary team according to protocol recommendations and local practice (“Supplemental information”).

### Follow-up and outcome

Neurological outcome was assessed at hospital discharge according to the Cerebral Performance Category (CPC) scale^21,22^: CPC 1—no neurological deficit; CPC 2—mild to moderate dysfunction; CPC 3—severe dysfunction; CPC 4—coma; and CPC 5—death. CPC scores of 1 and 2 are considered as ‘favorable’ outcomes and a CPC 3–5 ‘unfavorable’ outcomes.

### Statistical methods

Statistical analysis was performed using Stata V.16 (StataCorp. 2019. Stata Statistical Software: Release 16. College Station, TX: StataCorp LLC). For patient characteristics, results were expressed as counts and proportions, median with IQR or mean ± SD, as appropriate. Unpaired t-tests or Mann–Whitney U-tests were applied for unpaired comparisons of numerical variables. Chi-square or Fischer's exact test was applied to examine differences between categorical variables. Dynamic changes of MD variables were analyzed by longitudinal data analysis, using linear mixed models. Repeated measurements of MD variables on the same patient over time were taken into account by including a random intercept for each patient. Missing values, assumed to be missing at random, were handled by the mixed model fitting procedure. As this study was intended as a feasibility study, we decided not to correct for multiple testing. Associations between MD variables, ischemic periods, duration of cerebral desaturation, and neurological outcome were assessed with chi-square-test and logistic regression. Statistical comparison of secondary episodes of ischemia and cerebral desaturation between outcome groups was performed by utilizing the non-parametric Mann–Whitney U-test, and associations between peak values of MD variables and outcome groups were investigated by applying t-test. A p-value < 0.05 was considered as statistically significant.

## Results

### Patients

Eighteen unconscious patients with sustained ROSC, admitted to the intensive care unit following OHCA, were included in the study. The two outcome groups had similar baseline characteristics (Table 1).

**Table 1:** Baseline characteristics according to neurological outcome at hospital discharge.

Characteristic	Favorable outcome group (CPC 1–2) N = 5	Unfavorable outcome group (CPC 3–5) N = 13
**Demographic characteristics**		
Age (years)	56 ± 11	69 ± 11
Male sex—no. (%)	3 (60)	7 (58)
**Medical history—no. (%)**		
Chronic heart failure	2 (40)	3 (23)
Ischemic heart disease	3 (60)	3 (23)
Arterial hypertension	2 (40)	7 (54)
Previous stroke	1 (20)	2 (17)
Diabetes mellitus	1 (20)	4 (31)
Previous percutaneous coronary intervention	3 (60)	2 (15)
**Neurological function before cardiac arrest**		
Normal, CPC score 1^a^	4 (80)	13 (100)
Some disability, CPC score 2	1 (20)	0 (0)
**Characteristics of the cardiac arrest**		
Witnessed cardiac arrest—no. (%)	5 (100)	12 (92)
Bystander performed CPR—no. (%)	5 (100)	11 (85)
First monitored rhythm—no. (%)		
Shockable rhythm	5 (100)	9 (69)
Time from cardiac arrest to event (min)		
Start of basic life support, median (IQR)	2 [1, 2]	5 [1–8]
Start of advanced life support, median (IQR)	8 [8–15]	10 [8–14]
Return of spontaneous circulation, median (IQR)	20 [15–36]	22 [19–35]
**Clinical characteristics on admission**		
First measured body temperature (°C)	35.2 ± 0.8	35.4 ± 1.3
Glascow Coma Scale score^b^, median (IQR)	3 [3–3]	3 [3–3]
Pupillary reflex present—no. (%)	2 (40)	4 (31)
Serum pH	7.25 ± 0.05	7.18 ± 0.13
Serum lactate (mmol/l)	6.0 ± 3.1	7.4 ± 3.8
Circulatory shock—no. (%)^c^	2 (40)	6 (46)
ST-segment elevation myocardial infarction—no. (%)	3 (60)	6 (46)

Plus–minus values are means ± SD.

*CPC* cerebral performance category, *AMI* acute myocardial infarction, *CPR* cardiopulmonary resuscitation, *IQR* interquartile range.

^a^CPC score: 1, alert, able to work and lead a normal life; 2, moderate cerebral disability and sufficient cerebral function for part-time work; severe cerebral disability, dependent on others, and impaired brain function; 4, coma and vegetative state; 5, dead or certified brain dead.

^b^Scores on the Glasgow Coma Scale range from 3 to 15, with lower scores indicating reduced levels of consciousness.

^c^Circulatory shock was defined as a systolic blood pressure of less than 90 mmHg for more than 30 min.

### ICU

The median ICU length of stay (LOS) for outcome groups CPC 1–2 and CPC 3–5 were 72 [67–87] and 95 [72–144] h, respectively, and related sedation time for the groups was 25 [24–26] and 34 [26–44] h. The two outcome groups had similar hemodynamic and metabolic characteristics at ICU admission. During ICU stay, there were no overall difference in MAP (p > 0.45, when using mixed effects models), despite a significant difference in total norepinephrine use between outcome groups (p = 0.03) (Table 2). Additional main regulators of cerebral blood flow remained stable and within normal therapeutic range during ICU stay with no difference in CI, PaO_2_ and PaCO_2_ at any time points between outcome groups. Comprehensive results for neurological prognostication are displayed in Table 2.

**Table 2:** Post-resuscitation care data.

ICU monitoring	Favorable outcome group (CPC 1–2) N = 5	Unfavorable outcome group (CPC 3–5) N = 13
**Hemodynamics**		
MAP (mmHg)		
Day 1	74 ± 8	71 ± 7
Day 2	76 ± 12	74 ± 10
Day 3	81 ± 11	75 ± 10
CI (l/min/m^2^)		
Day 1	2.1 ± 0.4	2.2 ± 0.5
Day 2	2.9 ± 0.7	3.0 ± 0.7
Day 3	3.5 ± 0.3	3.1 ± 0.6
Average serum lactate (mmol/l)		
Day 1	1.7 ± 1.2	2.5 ± 2.3
Day 2	1.3 ± 0.6	1.6 ± 0.7
Day 3	0.9 ± 0.2	1.5 ± 0.7
Total vasopressor dose during ICU stay, median (IQR)		
Norepinephrine (mg)	7.9 [2.8–10.2]	**23.5 [15.8–28.1]**
Dopamine (mg)	277 [226–319]	575 [246–629]
**Respiratory**		
PaO_2_ (kPa)		
Day 1	15.5 ± 5.0	14.7 ± 4.1
Day 2	12.2 ± 3.9	12.8 ± 2.6
Day 3	11.0 ± 2.2	12.3 ± 2.1
PaCo_2_ (kPa)		
Day 1	5.6 ± 0.7	5.6 ± 1.1
Day 2	5.5 ± 0.9	5.2 ± 0.9
Day 3	5.2 ± 1.0	5.2 ± 0.8
**Cerebral**		
Mean INVOS—(rSO_2_%)		
Day 1	65 ± 9	68 ± 8
Day 2	74 ± 8	72 ± 7
Day 3	–	68 ± 9
Cerebral desaturation—% of total INVOS monitoring^a^	1.0% (n = 2)	0.6% (n = 2)
**Prognostication**		
Prognostication—no./total no. (%)	1/5 (20%)	9/12 (75%)
Time from OHCA to prognostication (h)	41 [41–41]	80 [75–94]
Myoclonic seizures—no./total no. (%)	1/5 (20%)	8/13 (62%)
CT, generalized edema—no./total no. (%)	0/5 (0%)	7/12 (58%)
EEG, highly malignant pattern—no./total no. (%)^b^	0/5 (0%)	7/12 (58%)
SSEP, bilaterally absent N20-respons—no./total no. (%)	0/5 (0%)	2/4 (50%)
**Jugular bulb microdialysis**		
Time from ROSC to microdialysis measurement (min)	310 [255–365]	300 [230–400]
Microdialysis time (hours)	40 [36–41]	72 [49–91]
Metabolic pattern < 24 ICU hours after ROSC		
Ischemia^c^	13% (13 h) n = 3	20% (45 h) n = 6
Mitochondrial dysfunction^d^	38% (36 h) n = 5	46% (110 h) n = 13

Plus-minus values are means ± SD. Bold text indicates a statistically significant difference with a p-value less than 0.05.

*N* number of patients, *MAP* mean arterial pressure, *CI* cardiac index, *INVOS* in-vivo optical spectroscopy (regional oxygen saturation), *OHCA* out-of-hospital cardiac arrest, *CT* computed tomography, *SSEP* somatosensory-evoked potentials, *EEG* electroencephalogram, *MD* microdialysis.

^a^Cerebral desaturation defined as rSO_2_ < 50%.

^b^EEG, highly malignant pattern defined as suppressed background without discharges, suppressed background with continuous periodic discharges; burst-suppression.

^c^Microdialysis verified ischemia defined as LPR > 16 and pyruvate < 70 µmol/l. Percentage (%) of total MD monitoring during the first 24 ICU hours.

^d^Microdialysis verified mitochondrial dysfunction defined as LPR > 16 and pyruvate > 70 µmol/l. Percentage (%) of total MD monitoring time during the first 24 ICU hours.

### Outcome

At hospital discharge a favorable outcome was observed in 28% patients, while 72% had an unfavorable outcome. Overall mortality during hospital stay was 61% due to primarily severe anoxic brain injury and withdrawal of life sustaining therapy. For detailed outcomes results, see Table 1 in “Supplementary information”.

### Comparison of biochemical variables monitored in arterial and jugular venous blood

JBM was initiated within approximately 4–6 h from ROSC in both outcome groups. Median monitoring times were 40 h (IQR 36–41) for CPC 1–2 and 72 h (IQR 49–91) for CPC 3–5. CT scans documented a correct positioning of JBM catheters in all patients. There were no observed complications related to the MD technique, and only a few MD artery catheters were associated with temporary malfunction due to clotting.

Figure 1 compares the changes over time in arterial and jugular blood for patients in the CPC 3–5 group regarding the biochemical variables (lactate, pyruvate, glycerol, glutamate, glucose), and the calculated LP ratio. The difference between time-averaged means of LP ratio, lactate, pyruvate, glycerol and glutamate were significant (p < 0.02) during the periods indicated in Fig. 1. JBM showed significantly elevated levels of glycerol compared to systemic MD in the first 50 h after ROSC (Fig. 1). In the late post-resuscitation period JBM, glutamate concentration was significantly higher than the arterial level (Fig. 1). Detailed results are given in Table 3.

**Table 3:** Time-averaged mean MD variables obtained in jugular bulb compared to artery in patients with unfavorable outcome.

Time from ROSC (h)	LP ratio		Lactate (mM)		Pyruvate (µM)	
	Jugular bulb	Artery	Jugular bulb	Artery	Jugular bulb	Artery
12	**25 [18–40]**	24 [16–32]	**2.6 [2.0–3.8]**	2.1 [1.3–3.1]	114 [56–165]	89 [60–173]
24	17 [14–21]	19 [15–29]	1.8 [1.4–2.8]	1.9 [1.3–2.8]	**113 [87–139]**	97 [50–121]
36	14 [12–19]	21 [14–29]	1.8 [1.3–2.6]	1.6 [1.2–2.5]	**120 [100–145]**	88 [53–115]
48	14 [12–15]	17 [14–22]	1.7 [1.4–2.4]	1.6 [1.3–2.2]	**126 [100–164]**	99 [66–148]
60	15 [12–17]	17 [14–20]	**1.6 [1.3–2.4]**	1.4 [1.1–1.9]	**123 [92–173]**	73 [62–127]
72	15 [12–17]	18 [15–22]	**1.5 [1.3–2.2]**	1.4 [1.1–1.8]	**112 [95–142]**	71 [52–118]
84	16 [14–18]	17 [15–20]	**1.7 [1.4–2.5]**	1.3 [1.0–1.7]	**113 [80–160]**	71 [52–123]
96	15 [13–17]	16 [15–18]	**1.8 [1.7–2.1]**	0.6 [0.5–1.7]	**131 [116–135]**	34 [30–108]

Time from ROSC (h)	Glucose (mM)		Glycerol (µM)		Glutamate (µM)	
	Jugular Bulb	Artery	Jugular Bulb	Artery	Jugular Bulb	Artery
12	7.2 [5.8–9.7]	8.1 [6.4–9.4]	**186 [118–279]**	157 [95–230]	58 [43–97]	63 [37–103]
24	6.5 [5.4–7.8]	6.6 [5.8–7.9]	**220 [142–258]**	131 [90–178]	52 [38–63]	48 [30–70]
36	6.6 [5.8–8.1]	6.1 [5.1–7.4]	**165 [132–219]**	115 [88–159]	47 [33–71]	50 [23–69]
48	7.6 [6.0–9.1]	7.4 [5.6–8.7]	**108 [77–143]**	88 [68–126]	53 [25–71]	33 [17–71]
60	7.9 [6.5–9.0]	7.3 [5.9–9.3]	81 [61–102]	69 [50–93]	56 [24–71]	29 [19–76]
72	7.8 [6.2–8.8]	8.3 [6.1–9.6]	70 [55–96]	72 [46–117]	**54 [28–66]**	27 [18–70]
84	7.8 [6.6–9.4]	7.8 [6.1–8.7]	65 [53–91]	**87 [62–134]**	60 [55–66]	54 [21–70]
96	8.3 [7.2–8.7]	8.3 [8.1–10.5]	69 [51–76]	**160 [36–168]**	**60 [51–86]**	80 [12–87]

Data are expressed as median (interquartile range). LP ratio: lactate/pyruvate ratio. Difference between time-averaged means of MD variables (in intervals of 12 h) of the jugular bulb and the arterial blood was assessed using mixed effects models. Hourly samples were analyzed separately, and total samples were averaged for intervals of 12 h. p < 0.05 is highlighted with bold.

In patients with favorable outcome, differences between time-averaged mean JBM variables and corresponding systemic values were statistically non-significant, except for glycerol (Table 2, “Supplementary information”).

### Cerebral metabolic patterns in outcome groups

The LP ratio in jugular blood remained elevated (> 16) during the first 20 h in both groups of patients (Fig. 2). After 20 h, an almost complete normalization of the LP ratio was observed. However, the cerebral level of lactate remained high in the CPC 3–5 group (mean level > 2.7 mM), and was paralleled by a marked increase in pyruvate. Based on the biochemical definitions above six patients with unfavorable outcome exhibited ongoing secondary ischemia during altogether 45 h (20%) of the first 24 h of MD monitoring. In the favorable group, three patients displayed a pattern of ischemia during altogether 13 h (13%) (Table 2). During this period, biochemical signs of mitochondrial dysfunction was noticed in 46% and 38% of the time in patients with unfavorable outcome (n = 13) and favorable outcome (n = 5) (Table 2), respectively. No significant difference between the outcome groups related to the extent of ischemia (p = 0.92) and mitochondrial dysfunction (p = 0.46) where observed, when applying mixed effects logistic regression.

### JBM variables in relation to critical clinical episodes

JBM levels in patients with myoclonic seizures (n = 9) were not significantly different from other patients, except for slightly lesser lactate levels during the first 12 h (p = 0.012). EEG-verified epileptiform activity was only registered in three patients with unfavorable outcomes, without difference in JBM levels. Regression analysis showed a significant negative correlation between MAP and corresponding jugular bulb lactate in patients with poor outcomes. For a one-unit increase in MAP (mmHg), cerebral lactate changed by − 0.011 mM (p = 0.005). However, no extreme JBM variables were seen during hypotensive periods. For the entire cohort, hypotensive periods with critical MAP < 60 mmHg were observed for only 23 h (1.9%) during ICU stay. Thus, analysis of hypotensive periods did not result in significant JBM correlations.

### Association between JBM and neurological outcome

The time course for changes in all measured biochemical variables are illustrated for outcome groups CPC 1–2 and CPC 3–5 in Fig. 2. No significant difference between time-averaged means of JBM variables during post-resuscitation care was obtained (p > 0.1). However, there was a clear tendency towards systematic negative coefficients in JBM variables when comparing CPC 1–2 to the CPC 3–5 group. In patients with unfavorable outcome, cerebral venous lactate remained high with mean and peak venous lactate level > 2.7 mM and 6.6 mM, respectively. The increased lactate level was paralleled by a marked and lasting increase in pyruvate (mean and peak values above 137 µM and 268 µM, respectively). The latter correlated significantly to unfavorable outcome (p = 0.02).

### Regional cerebral desaturation

**Figure 3:**
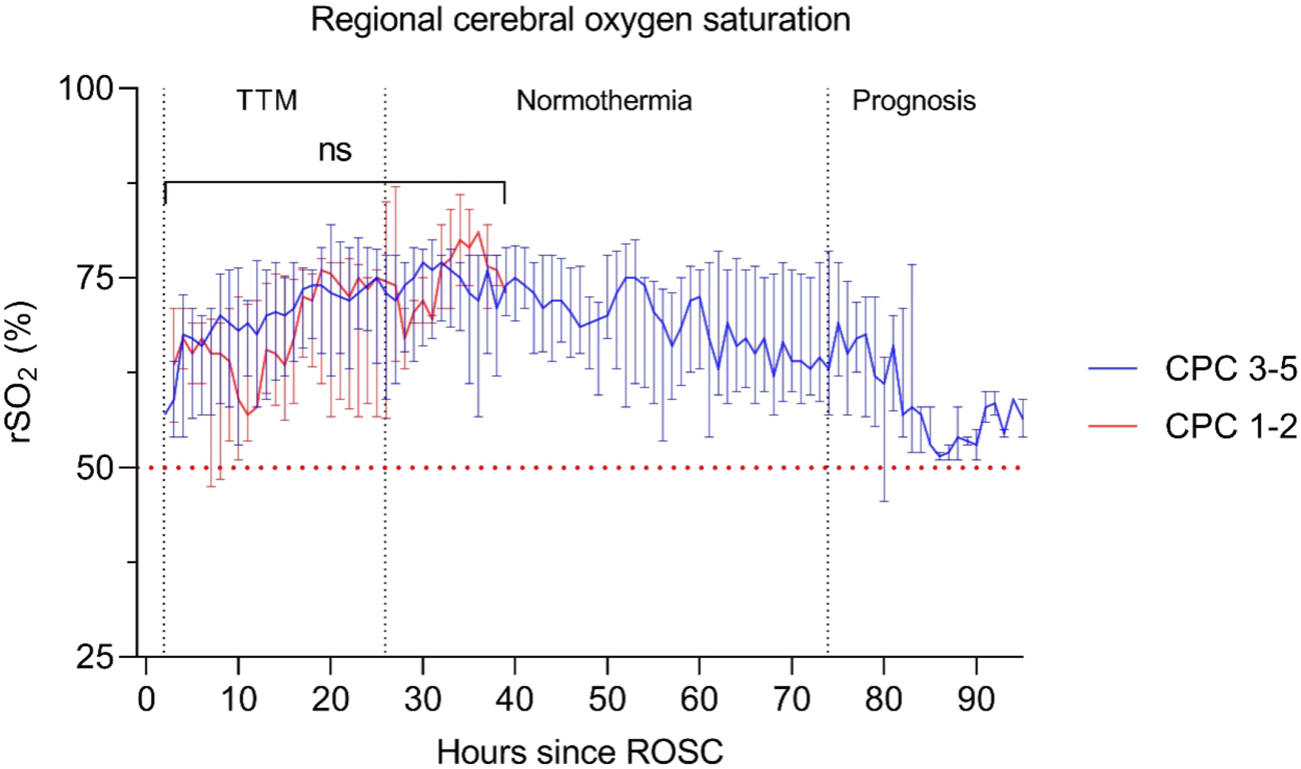
Median (IQR). Regional cerebral oxygen saturation (rSO_2_) during post-resuscitation care in patients with favorable (CPC 1–2) and unfavorable (CPC 3–5) outcome. No significant (ns) differences between outcome groups were observed. The dotted red line indicates cerebral desaturation threshold rSO_2_ < 50%.

Two patients in each CPC group showed minimal transient signs of cerebral desaturation with rSO_2_ < 50% in 1.0% (CPC 1–2) and 0.6% (CPC 3–5) of total INVOS monitoring period (Fig. 3, Table 2). No differences in rSO_2_ between outcome groups were observed at any time point, using mixed effects models for statistical evaluation (Fig. 3). There was no correlation between JBM-verified ischemic periods and cerebral desaturation.

### Correlation between systemic blood lactate and MD arterial lactate

The correlation and agreement between systemic blood lactate (Lac_sys_) and MD arterial lactate (Lac_MD-Art_) was calculated to evaluate the accuracy of the MD technique. A highly significant correlation with r = 0.73 and coefficient at 0.82 [0.75–0.89] (p < 0.0001) was obtained. Bland–Altman statistics showed an average bias for Lac_sys_ of 0.18 mM higher than Lac_MD-Art_ with the 95% limits of agreement ranging from − 0.75 to 1.11 (Fig. 3A, “Supplementary information”).

## Discussion

Contrasting to previous studies utilizing JBM cardiac arrest causes complete ischemia and compromised energy metabolism in all body tissues^13,16^. To document that the biochemical variables measured in cerebral venous blood after ROSC reflected their intracerebral levels, MD catheters were placed in a peripheral artery for systemic reference, as well as in the jugular bulb.

As shown in Fig. 1 the concentrations of glycerol, lactate and pyruvate were significantly higher in jugular than in arterial blood during most of the studied period supporting the hypothesis that JBM may be used to monitor cerebral energy metabolism after OHCA. However, JBM-detection of isolated cerebral metabolic perturbation is dependent on a certain degree of brain injury, as shown in patients with poor outcome.

Further, a strong correlation between intracerebral MD lactate and JBM lactate measurements, was described in patients suffering from aneurismal subarachnoid hemorrhage^16^. However, the measured levels of the variables do not represent their true interstitial concentrations: transport limitation across the blood–brain barrier (BBB), relative recovery of the microdialysis membrane and estimated imprecision (approximately 3–7%) of the analytical techniques all contribute to this fact^23^. A strong correlation between systemic blood lactate and MD arterial lactate was obtained. In future JBM studies, systemic lactate may replace invasive MD arterial lactate monitoring as a reference.

Under normal conditions, the arteriovenous difference for glucose is approximately 0.5 mM. This small difference would not be quantitatively confirmed by the present microdialysis and analytical techniques explaining the absence of difference between arterial and venous glucose concentrations (Fig. 1, Table 3)^23^.

Glycerol concentration in adipose tissue and blood increases markedly during stress and decreases in the post-stress period^24^. In the present CPC 3–5 group, a slight increase of arterial glycerol level was observed initially but during most of the studied period glycerol was within normal range (Fig. 1). In venous jugular blood, glycerol levels were significantly higher than in arterial blood during the period 0–48 h (Fig. 1, Table 3). An increase in intracerebral glycerol concentration is a marker of degradation of phospholipids in cellular membranes, but the BBB permeability for glycerol is limited^25^. Accordingly, the significant increase of glycerol in jugular venous blood is interpreted as reflecting degradation of cerebral cellular membranes possibly in combination with an increased BBB permeability.

The intact BBB has a limited permeability for glutamate. Normal glutamate concentration in blood has been reported to be 30–80 µM at a cerebral interstitial concentration of < 1 µM^26^. In the present study, median jugular venous glutamate level was within normal range during the study period (Fig. 1).

A sudden, complete interruption of CBF is instantaneously reflected in a shift in cytoplasmic redox state and a marked increase in cerebral LP ratio^27^. Within a few minutes, variables reflecting cerebral energy state are completely depleted^27^. If CBF is adequately restored, reperfusion after 30 min of global cerebral ischemia, results in an almost complete normalization of cerebral LP ratio within 90 min^28^. However, the level of lactate remains high and is paralleled by a marked increase in pyruvate^28^. Transient cerebral ischemia is known to cause mitochondrial dysfunction and persistently altered mitochondrial function has been documented after apparently successful resuscitation in experimental cardiac arrest^29–31^.

For practical reasons it was not possible to obtain biochemical MD data until approximately 4–7 h after ROSC (Table 2). In spite of this delay, cerebral ischemia was diagnosed in altogether nine patients during the initial 24 h of MD. The observation contrasts to experiences in experimental studies presented above. In these studies, biochemical signs of remaining ischemia were not observed 90 min after recirculation^28,32^. The finding reveals that under clinical conditions prolonged periods of impaired cerebral perfusion are frequent after ROSC. Cerebral reperfusion after OHCA is complex and there is a lack of data regarding the first hours after ROSC^33^. The pattern of ischemia and compromised energy metabolism would probably have been even more pronounced if earlier monitoring were possible. rSO_2_ is thought to reflect CBF, and a recent study found a moderate correlation between rSO_2_ and cerebral perfusion pressure^34^. The notion that delayed cerebral hypoperfusion is important during the first hours after ROSC was recently supported in a study measuring cerebral oxygenation^35^. In accordance with other studies, no significant difference in rSO_2_ between outcome groups was observed^36^. The JBM detection of ongoing secondary brain ischemia may potentially aid clinicians in providing individualized brain resuscitation strategies that prevent secondary brain injury and lead to improved survival and neurological outcomes after cardiac arrest, e.g. increasing blood pressure, optimizing cardiac output and modifying PaO_2_ and PaCO_2_.

Increased levels of lactate and pyruvate were observed during the entire monitoring period in the CPC 3–5 group and was more pronounced than in the CPC 1–2 group (Fig. 2). The interpretation of mitochondrial dysfunction, is supported by the study by Zhang et al. describing significant increases in [18F]-FDG uptake in the brain of post-CA animals^37^. The discrepancy between the two groups indicates that energy metabolism was initially more compromised in CPC group 3–5 (Fig. 2, Table 2).

However, the biochemical pattern interpreted as mitochondrial dysfunction does not necessarily prove malfunctioning mitochondria. A similar metabolic pattern is observed when cerebral energy requirements exceed mitochondrial capacity for oxidative metabolism (e.g., severe epileptic seizures; c.f. myoclonic seizures Table 2) and in hypoxic hypoxia^38,39^. In this study, JBM levels in patients with myoclonic seizures were not significantly different from other patients. Myoclonus can indicate irreversible brain damage or a benign clinical finding not related to poor outcome. Nonetheless, myoclonic seizures did not seem to affect global oxidative cerebral metabolism. Interestingly, an elevation in the jugular venous bulb oxygen tension-brain oxygen tension gradient was recently described after ROSC^40^. This elevation was not modulated by changes in cerebral perfusion pressure, which indicates a diffusion limitation of oxygen delivery. Accordingly, there might be various ways of treating the pattern of mitochondrial dysfunction observed after ROSC.

The parallel increase in lactate and pyruvate at an unchanged LP ratio observed in the CPC 1–2 group during the time periods 28–40 h and after 48 h reflects the biochemical pattern during increased cerebral metabolic rate induced by reduced sedation, arousal, and stress reaction^19,41^. These fluctuations in lactate and pyruvate are expected in the awakening brain and decrease the possibility of obtaining a statistically significant difference between the two outcome groups.

## Limitations

The number of patients in this feasibility study was limited and the clinical value of the technique should be evaluated in a larger study. The study population consisted of OHCA patients exclusively with presumed cardiac cause due to the clinical setup in our department. During the first 12 h the systemic lactate was statically higher in the CPC 3–5 group compared to the CPC group 1–2 (p = 0.042) which could affect the differences in elevated JBM lactate in patients with poor outcome. In the present clinical situation, global brain ischemia would be expected and lateralization in cerebral venous drainage affecting jugular bulb sampling is probably of limited importance. The normal range of the biochemical variables measured, and classification of neuro-metabolic patterns, are based on minor patient material. Outcome assessors were not blinded to JBM data increasing the risk of bias. Studies comparing JBM verified ischemia/mitochondrial dysfunction to other global measures of cerebral metabolism/ischemia, e.g., positron emission tomography (PET-CT), are warranted. Combined JBM and CBF measurements (e.g. brain ^15^O-H_2_O-PET CT or Xenon-enhanced CT) might provide additional insights in the dynamic pathogenic mechanism related to secondary brain injury described in the study. The assessment of global cerebral metabolism was delayed after ROSC due to logistic reasons, and biochemical-signals would probably have been more pronounced if initiated earlier. Microdialysis is an established technique for experimental and clinical studies. Its use in routine intensive care has, however, been limited by the fact that microvials must be transported from the patient to the analyzer at regular intervals (hourly). A system for online MD, biochemical analysis and display (lactate, pyruvate, glucose) has recently been commercially available (MD System Loke, MDialysis AB, Stockholm, Sweden). This online technique would facilitate biochemical monitoring within the first hours after ROSC.

## Conclusions

Bedside microdialysis monitoring of the cerebral drainage in the jugular bulb is feasible and safe during post-resuscitation care. Microdialysis results indicated that cerebral metabolic parameters could be distinguished from systemic parameters in patients with poor outcomes, and may be used in comatose OHCA patients to assess global cerebral energy metabolism. However, detection of isolated cerebral metabolic perturbations is dependent on a certain degree of brain injury. Impaired cerebral perfusion and neuro-metabolic signs of ischemia and mitochondrial dysfunction are frequent and long-lasting after ROSC and more pronounced in patients with unfavorable outcomes. The technique might be used for real-time brain injury detection and treatment titration early after ROSC to improve outcomes. However, the clinical value must be evaluated in a more extensive study, why we are conducting a randomized control trial addressing the effect of higher MAP on global cerebral metabolism in 60 comatose patients resuscitated from OHCA.

## Supplementary Information

Supplementary Information.
