# Supplementary material for: Bedside microdialysis for detection of early brain injury after out-of-hospital cardiac arrest
Source: Sci Rep. 2021 Aug 5;11:15871. doi: 10.1038/s41598-021-95405-9 (PMC8342553; doi:10.1038/s41598-021-95405-9)
Supplement: Supplementary file 1 — Supplementary Information. [file 41598_2021_95405_MOESM1_ESM.docx]

Supplementary information

**Jugular bulb catheter**

The correct positioning of the jugular bulb catheter tip (red arrow) was confirmed on cranial computed tomography (CT) scan; see Figures 1 and 2 for examples.

Figure 1


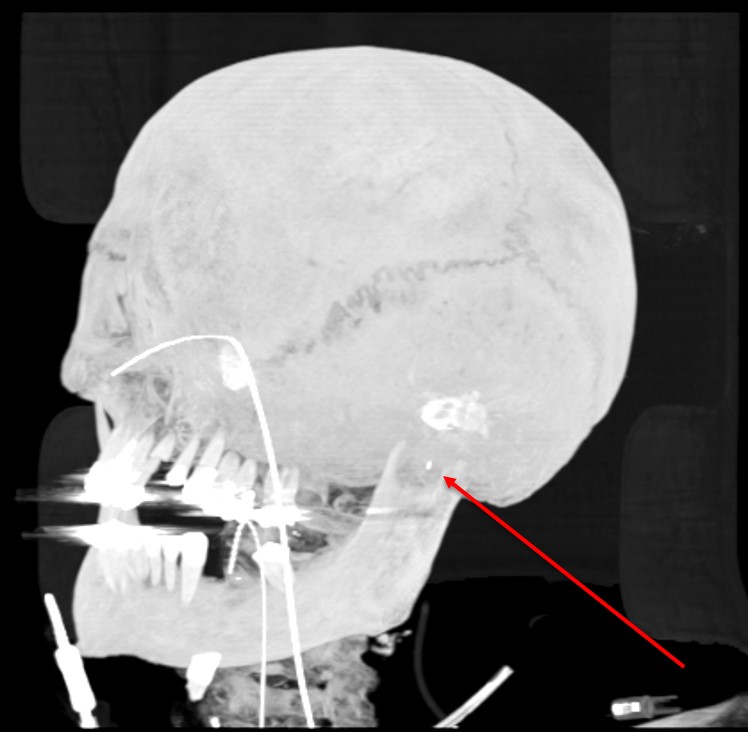


CT scan with 3D reconstruction documented a correct positioning of the jugular bulb catheter tip (red arrow). The image has been downloaded from the GE Web Pacs database with permission to use in the article.

Figure 2


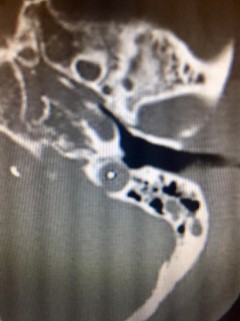


CT scan in the axial plane documented a correct positioning of the jugular bulb catheter tip (red arrow). The image has been downloaded from the GE Web Pacs database with permission to use in the article.

**Withdrawal of life supporting therapies (WLST)**

In an unconscious patient with Glasgow Motor Score of ≤3 at ≥72 h from ROSC, in the absence of confounders, poor outcome was considered when two or more of the following predictors were present: no pupillary and corneal reflexes at ≥72 h, bilaterally absent N20 SSEP wave at ≥24 h, highly malignant EEG at ≥24 h (suppressed background ± periodic discharges or burst-suppression), status myoclonus ≤72 h (continuous and generalized myoclonus persistent for 30 minutes or more), or a diffuse and extensive anoxic injury on brain CT. If a decision of WLST was made, the time point and the main reasons for withdrawing life-supporting therapies were recorded.

**Table 1. Outcomes**

| **Outcome** | **Favorable outcome group (CPC 1-2)**  **N = 5** | **Unfavorable outcome group (CPC 3-5)**  **N = 13** |
| --- | --- | --- |
|  | ***no./total no. (%)*** | |
| **Deaths at the end of study** | 0/5 (0%) | 11/13 (85%) |
| Location of death  ICU  Ward |  | 7/11 (64%)  4/11 (36%) |
| Cause of death  Cerebral  Hemodynamic |  | 10/11 (91%)  1/11 (9%) |
| **Neurologic function at hospital discharge** |  |  |
| CPC Category*  1  2  3  4  5 | 2/5 (40%)  3/5 (60%) | 2/13 (15%)  11/13 (85%) |

Text Table 1. Abbreviations: ICU, intensive care unit; CPC, Cerebral Performance Category. *CPC score: 1, alert, able to work and lead a normal life; 2, moderate cerebral disability and sufficient cerebral function for part-time work; severe cerebral disability, dependent on others, and impaired brain function; 4, coma and vegetative state; 5, dead or certified brain dead.

**Table 2. Time-averaged mean MD variables obtained in jugular bulb compared to artery in patients with favorable outcome**

| Time from ROSC | LP ratio | | Lactate mM | | Pyruvate µM | |  |
| --- | --- | --- | --- | --- | --- | --- | --- |
|  | Jugular Bulb | Artery | Jugular Bulb | Artery | Jugular Bulb | Artery |  |
| 12 h | 19 [16-38] | 18 [14-21] | **1.8 [1.5-3.7]** | 1.6 [1.3-2.2] | 90 [76-103] | 88 [73-117] |  |
| 24 h | 15 [13-27] | 22 [13-32] | 1.3 [1.2-1.8] | 1.6 [1.0-1.9] | 81 [61-98] | 68 [56-95] |  |
| 36 h | 16 [14-20] | 16 [13-35] | 2.1 [1.4-2.7] | 2.2 [1.6-2.9] | 101 [89-144] | 100 [68-153] |  |
| 48 h | 13 [12-15] | **24 [13-31]** | 1.1 [1.0-1.9] | 1.6 [1.4-1.9] | 91 [80-120] | 71 [53-114] |  |
| 60 h | 10 [10-16] | 22 [15-27] | 1.5 [1.2-1.6] | 2.0 [1.8-2.1] | 121 [100-129] | 98 [72-121] |  |
| 72 h | - | - | - | - | **-** | - |  |
| 84 h | - | - | - | - | **-** | - |  |
| 96 h | - | - | - | - | **-** | - |  |
|  | | | | | | |  |
| Time from ROSC | Glucose mM | | Glycerol µM | | Glutamate µM | |  |
|  | Jugular Bulb | Artery | Jugular Bulb | Artery | Jugular Bulb | Artery |  |
| 12 h | 7.1 [6.3-8.0] | 6.6 [5.3-7.6] | 186 [129-229] | 185 [142-236] | 59 [49-95] | 55 [48-79] |  |
| 24 h | 6.7 [6.1-7.6] | 6.7 [3.8-8.2] | **168 [141-242]** | 151 [107-173] | 58 [39-73] | 63 [43-72] |  |
| 36 h | 6.9 [5.7-7.7] | 5.0 [3.9-7.9] | **160 [92-196]** | 120 [88-163] | 64 [55-78] | 60 [43-76] |  |
| 48 h | 5.1 [4.9-5.8] | 5.2 [4.8-6.1] | 57 [42-84] | 69 [57-87] | 58 [50-60] | 54 [50-60] |  |
| 60 h | 5.9 [5.3-6.1] | 5.5 [4.6-6.0] | 51 [45-89] | 68 [64-85] | 60 [58-64] | 55 [52-65] |  |
| 72 h | - | - | - | - | **-** | - |  |
| 84 h | - | - | - | **-** | - | - |  |
| 96 h | - | - | - | **-** | **-** | - |  |
|  | | | | | | |  |

Text Table 2. Data are expressed as median (interquartile range). LP ratio: lactate/pyruvate ratio. Difference between time-averaged means of MD variables (in intervals of 12 hours) of the jugular bulb and the arterial blood was assessed using mixed effects models. Hourly samples were analyzed separately, and total samples were averaged for intervals of 12 hours. P<0.05 is highlighted with bold. (-) indicates no measured microdialysis samples due to awake state.

**Figure 3A.**

Bland-Altman plot showing the correlation between systemic blood lactate and microdialysis arterial lactate:

The correlation and agreement between systemic blood lactate (Lac_sys_) and MD arterial lactate (Lac_MD-Art_) was calculated to evaluate the accuracy of the MD technique. A highly significant correlation with r=0.73 and coefficient at 0.82 [0.75-0.89] (p<0.0001) was obtained. Bland-Altman statistics showed an average bias for Lac_sys_ of 0.18 mM higher than Lac_MD-Art_ with the 95% limits of agreement ranging from −0.75 to 1.11.

**Figure 3B.**

Bland-Altman plot showing the correlation between systemic blood glucose and microdialysis arterial glucose:

The correlation and agreement between systemic blood glucose and MD arterial glucose was calculated to evaluate the accuracy of the MD technique. A highly significant correlation with r=0.60 and coefficient at 0.86 [0.73-0.99] (p<0.0001) was obtained. Bland-Altman statistics showed an average bias for systemic glucose of 0.14 mM higher than MD artery glucose
